# Acute fulminating massive pulmonary embolism treated with aggressive endovascular or surgical approach And ECMO

**DOI:** 10.1186/1749-8090-10-S1-A217

**Published:** 2015-12-16

**Authors:** Shih-Chen Tsai, Jung-Ming Yu, Su-Chin Tsao, Ying-Che Sun, Yi-Liang Wu, An-Hua Sun, Tsung-Po Tsai

**Affiliations:** 1 Department of Medical Education, Cathay General Hospital, 280 Renai Rd, Sec.4, Taipei, Taiwan; 2 Division of Cardiovascular Surgery, Chung Shan Medical University Hospital, Jianguo N Rd, South District, Taichung City, Taiwan

## Aims/Objectives

Massive pulmonary embolism (PE) is frequent lethal, but rapid diagnosis and aggressive therapy with endovascular or surgical thromboembolectomy and extracorporeal membrane oxygenation (ECMO) may be lifesaving. We reviewed our current cases to find that whether the aggressive surgical approaches is the best alternative or not.

## Method

Five female patients (age 23, 37, 58, 70 and 76, mean 52.8) were diagnosed as massive pulmonary embolism with either acute irreversible pulmonary failure or cardiac collapse (2 CPR) by Chest CT or pulmonary angiography. All patients required ECMO support in addition to 2 endovascular Angiojet aspiration or 1 surgical pulmonary thromboembolectomy. The duration for ECMO survivors were 1, 2 and 7 days, respectively. All patients required anticoagulation (3 heparin, 2 r-TPA, 3 Coumadin and 1 urokinase) to resolve the residual emboli.

## Results

One died from ECMO cannula insertion complication of massive retroperitoneal hematoma and bleeding and the other one died of multiorgan failure (MOF). Three were weaned from ECMO and was discharged in good condition at follow-up.

## Discussion/Conclusion

Aggressive endovascular or surgical pulmonary thromboembolectomy in combination with ECMO appears to have beneficial effects for massive pulmonary embolism with acute cardiopulmonary failure.

